# The economic value of targeting aging

**DOI:** 10.1038/s43587-021-00080-0

**Published:** 2021-07-05

**Authors:** Andrew J. Scott, Martin Ellison, David A. Sinclair

**Affiliations:** 1 Department of Economics, London Business School, London, UK; 2 Department of Economics, University of Oxford, Oxford, UK; 3 Harvard Medical School, Boston, MA USA

**Keywords:** Social sciences, Senescence, Ageing

## Abstract

Developments in life expectancy and the growing emphasis on biological and ‘healthy’ aging raise a number of important questions for health scientists and economists alike. Is it preferable to make lives healthier by compressing morbidity, or longer by extending life? What are the gains from targeting aging itself compared to efforts to eradicate specific diseases? Here we analyze existing data to evaluate the economic value of increases in life expectancy, improvements in health and treatments that target aging. We show that a compression of morbidity that improves health is more valuable than further increases in life expectancy, and that targeting aging offers potentially larger economic gains than eradicating individual diseases. We show that a slowdown in aging that increases life expectancy by 1 year is worth US$38 trillion, and by 10 years, US$367 trillion. Ultimately, the more progress that is made in improving how we age, the greater the value of further improvements.

## Abstract (web summary)

An economic analysis suggests that targeting aging offers potentially larger economic gains than eradicating individual diseases. Slowing aging to increase life expectancy by 1 year is worth US$38 trillion, and by 10 years, US$367 trillion.

## Main

Life expectancy (LE) has increased dramatically over the past 150 years^1^, although not all of the years gained are healthy. Analysis of the Global Burden of Disease dataset^2^ suggests that the proportion of life in good health has remained broadly constant, implying increasing years in poor health. Furthermore, the disease burden is shifting towards chronic non-communicable diseases, estimated to have caused 72.3% of deaths in the United States in 2016. The result is “a substantial part of life, and certainly most deaths, now occur in a period in the lifespan when the risk for frailty and disability increases exponentially.”^3^ As a consequence, there is a growing emphasis on ‘healthy aging’ and an emerging body of research focusing on the biology of aging (see refs. ^4,5^). According to another paper, “this era marks an inflection point, not only in aging research but also for all biological research that affects the human healthspan.”^6^

These developments pose a number of important questions. Is it preferable to make lives healthier by compressing morbidity, or longer by extending life? What are the gains from targeting aging itself, with its potential to make lives both healthier and longer? How does the value of treating aging compare to eradicating specific diseases? How will the economic value of these gains evolve over time? To answer these questions, we take an economic rather than biological perspective. Specifically, we use the value of statistical life (VSL) methodology to place a monetary value on the gains from longer life, better health, and changes in the rate at which we age^7–9^.

VSL models have two distinct advantages for our purposes. First, they are already used by government agencies to evaluate different policy measures and treatments, for example^10,11^. Second, as our model is based around optimizing economic agents, we can calculate not only the current gains from targeting aging but also how these gains will evolve in response to potential future changes in health and LE. The results reveal a distinctive feature of age-targeting treatments. Interactions between health, longevity, economic decisions and demographics create a virtuous circle, such that the more successful society is in improving how we age, the greater the economic value of further improvements.

## Results

Our economic model is based on that in ref. ^8^ and calibrated to current US data. In the model, individuals make choices about consumption, hours worked and leisure based on wage rates, interest rates, retirement age, and knowledge of remaining LE and likely future health. Changes in health or longevity lead to changes in these economic decisions, enabling us to estimate an individual’s willingness to pay (WTP) for these improvements. WTP is measured in US dollars and reflects the increase in VSL induced by improvements in health and longevity. VSL is the sum of the value of each remaining year of life, discounted to the present day and weighted by the survival rate. As the value of each year of life depends on health, consumption and leisure, the VSL incorporates both the quantity and quality of expected life remaining. Importantly, this means that the VSL is higher than an individual’s lifetime income; life is valuable in its own right because individuals value time, health and leisure.

The demographic data underpinning our analysis are: (1) a survival function^12^, in which mortality risk increases exponentially with age; (2) a health deficit function^13^, which also increases exponentially with age; and (3) the 2017 population structure and birth rates from the US Census Bureau. In our baseline calculations, LE and healthy life expectancy (HLE) at birth are 78.9 and 68.5 years, respectively, based on current US data. Following ref. ^14^, we set the average VSL of an adult aged between 25 and 65 years to US$11.5 million. Although our dollar WTP values are sensitive to the precise calibration of our model, the relative importance of different treatments for aging is not.

### Life extension (the Struldbrugg case)

We first focus on improving LE which, with reference to *Gulliver’s Travels*^15^, we refer to as the Struldbrugg case. Struldbruggs, both male and female, are born immortal but age normally, so live in continuously worsening health. In our simulations, we achieve this by reducing the rate at which mortality increases with age while holding unchanged the rate at which health declines. The result is an expansion of morbidity such that the ratio of HLE to LE deteriorates.

**Fig. 1:**
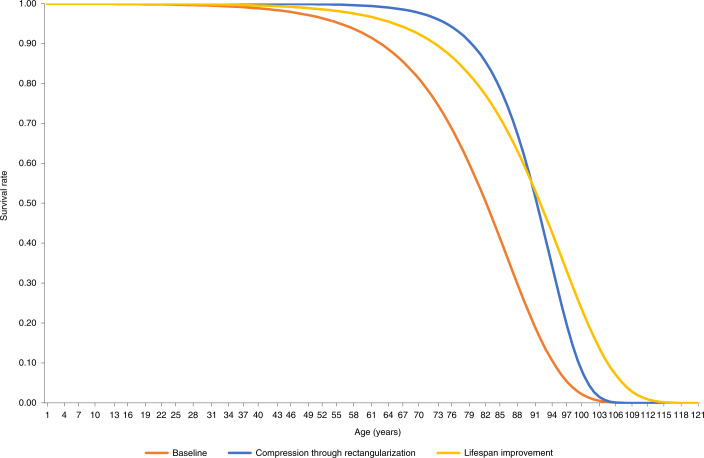
Survival functions under rectangularization and improvement in lifespan. Three different survival functions (probability of surviving from birth to different ages). Baseline is a standard Gompertz–Makeham survival function calibrated to 2019 US data. The rectangularization curve shows a survival function in which improvements in LE are achieved through compressing morbidity. The lifespan improvement curve shows a survival function in which improvements arise through elongation of the aging process. Source data

The WTP for LE increases depends on which years benefit from lower mortality. To provide consistency across simulations, we assume mortality is subject to a compensating effect^16^ whereby it reaches a rate *M* at age *T*. Under this specification, there are two ways to extend LE. The first is to rectangularize the survival function such that *M* and *T* are kept constant but mortality falls at all ages less than *T* while rising more rapidly at *T* (Fig. 1, blue survival function). The second involves increasing lifespan, such that mortality reaches *M* at higher values of *T*. In this case, survival rates decline more slowly, increasing the probability of living beyond *T* (Fig. 1, yellow survival function).

Table 1 shows the WTP for increases of 1 year in remaining LE through rectangularization versus improvements in lifespan, at ages 0, 20, 40, 60 and 80 years. The first row is the WTP for the initial (First) 1-year increase from our baseline; for example, the WTP at birth for the initial 1-year increase in remaining LE from 78.9 to 79.9 years via rectangularization is US$118,100, the WTP at age 60 for the first 1-year increase in remaining LE from 21.7 to 22.7 years through lifespan improvement is US$257,700. The subsequent rows (Second, Third, and so on) show the WTP for additional 1-year increases, so in the row starting ‘Tenth’, the WTP at age 20 shows an increase in remaining LE from 68.0 to 69.0 years, since by the tenth increment the remaining LE at age 20 has already risen to 68.0 years. Although 1-year increases in LE are convenient for presentational simplicity, each additional year of LE requires ever-larger proportional changes in mortality rates at older ages, as noted in ref. ^17^.

**Table 1:** WTP for 1-year increases in remaining LE (the Struldbrugg case)

	Age at which WTP is calculated (years)									
	0		20		40		60		80	
	R	L	R	L	R	L	R	L	R	L
First	118.1	96.5	171.0	141.5	232.0	199.4	285.6	257.7	312.2	288.1
Second	114.1	93.4	165.5	137.1	226.1	193.2	279.7	250.0	304.4	278.1
Third	110.0	90.4	160.1	132.7	220.0	187.2	273.8	242.1	298.0	268.5
Fourth	105.9	87.5	154.5	128.4	213.8	181.3	267.7	234.6	292.0	259.4
Fifth	101.8	84.6	148.9	124.3	207.4	175.6	261.5	227.4	286.0	250.7
Tenth	81.8	71.5	120.8	105.0	173.0	149.1	226.2	193.8	225.3	212.2
Twentieth		50.3		74.0		105.9		139.3		152.7
Thirtieth		35.0		51.6		74.3		98.8		109.5

WTP for the first, second and further 1-year increases in remaining LE at ages 0, 20, 40, 60 and 80 years. The LE remaining at these ages in the baseline simulation is 78.9, 59.0, 39.5, 21.7 and 8.4 years. Some values are missing because there is an upper limit to how much LE can be extended through rectangularization. Values are given in US$1,000. L, improvements in lifespan *T*; R, rectangularization.

Three results stand out from Table 1: the WTP for additional gains in LE diminishes as LE rises, it is greatest at older ages, and is higher under rectangularization. WTP diminishes as LE rises because the gains progressively accrue more in the future, which means they are discounted more and occur in years of poor health. The fact that WTP increases with age is also partly due to discounting (the old experience the benefits of extra LE sooner than the young) but mainly because the probability of reaching older ages and benefiting from the gains is increasing with age itself. Rectangularization is preferred because it concentrates increases in LE.

### Compressing morbidity (the Dorian Gray case)

We next hold LE fixed but improve the relationship between health and age. Under this scenario, which we refer to as the Dorian Gray^18^ case, HLE rises as a proportion of LE to create a ‘compression of morbidity’^19^. In the eponymous novel, Dorian Gray has a portrait painted and while the picture ages, Gray himself does not, retaining his health and looks until he dies. Following ref. ^20^, we assume morbidity is also subject to a compensating effect such that health declines to reach H* at age T*. Under rectangularization, gains are reflected in better health prior to T* but a faster deterioration around T*, whereas improvements in healthspan stretch the health function so it reaches H* at higher values of T*.

The results (Table 2) indicate that the WTP for improvements in HLE diminishes as HLE rises and also increases with age. As before, this reflects a combination of discounting and the higher probability of older individuals reaching even older ages. However, an additional force is at work in this case because as health improves in later life, individuals respond by allocating more consumption and leisure to these years, and gains in health at older ages become more attractive. This also explains why extending healthspan is eventually preferable to rectangularization.

**Table 2:** WTP for 1-year increases in remaining HLE (the Dorian Gray case)

	Age at which WTP is calculated (years)									
	0		20		40		60		80	
	R	H	R	H	R	H	R	H	R	H
First	242.0	216.3	377.0	328.5	472.7	429.7	570.8	536.9	692.5	653.8
Second	233.4	210.0	359.0	317.8	452.3	416.8	538.0	519.2	612.0	618.6
Third	224.1	203.8	341.0	307.6	432.6	404.6	513.4	503.7	588.7	598.8
Fourth	214.2	197.9	322.6	297.7	413.0	393.0	493.0	489.8	531.6	583.9
Fifth	203.7	192.1	303.8	288.3	393.1	381.9	474.0	477.1		
Tenth	136.6	165.3	197.0	245.7	230.0	331.6				

WTP for the first, second and further 1-year increases in remaining HLE at ages 0, 20, 40, 60 and 80 years. The HLE remaining at these ages in the baseline simulation is 68.5, 48.8, 30.4, 14.9 and 4.8 years. Values are given in US$1,000. Some values are missing because there is an upper limit to how much HLE can be extended through rectangularization or improvements in lifespan. H, improvements in healthspan T*; R, rectangularization.

Tables 1 and 2 show that the economic value of gains from an extra year of HLE always exceed those from an extra year of LE. An increase in LE in the Struldbrugg case provides additional years in which to enjoy lifetime consumption and leisure, but declining health makes this less appealing than the increase in health at each age under the Dorian Gray case. This preference for HLE over LE extends to preferring a full compression of morbidity. Even though the WTP for additional years of HLE decreases with further 1-year increases in remaining HLE (Table 2), it never falls below the WTP for the first increase in LE in Table 1. Individuals always prefer an extra year of HLE to adding an additional year to current US LE.

### Slowing aging (the Peter Pan case)

We now consider the WTP for slowing aging itself, which leads to simultaneous improvements in health and mortality. We assume aging occurs through the accumulation of biological damage, and that slowing aging lessens the pace at which health and mortality deteriorate with age. In the extreme case, where aging is not just slowed but eliminated, mortality and health become independent of age and the individual is ‘forever young’. We refer to this as the ‘Peter Pan’ case, after the play and novel^21^ about a boy who never grows old. To allow for a slowdown in aging we multiply chronological age *a* by a constant δ. For δ = 1, biological damage accumulates at its current rate but the lower δ is, the more slowly aging occurs and the greater the gap between biological and chronological age. The ‘forever young’ case is given by δ = 0.

In contrast to the Struldbrugg and Dorian Gray cases, WTP now consists of two components, one representing the gains in mortality and the other representing gains in health. Table 3 shows the total WTP for slowing down aging to achieve 1-year step increases in LE. Compared to Struldbrugg, Peter Pan has higher WTP because now both health and LE are increasing. The WTP for further delays in aging still declines but at a slower rate owing to the complementarities between health and longevity; that is, the higher the LE, the greater the WTP for an increase in health, and the better the health, the greater the WTP for improvements in LE.

**Table 3:** WTP for 1-year increases in remaining LE (the Peter Pan case)

	Age at which WTP is calculated (years)				
	0	20	40	60	80
First	178.7	262.6	333.9	378.5	380.2
Second	175.1	257.4	328.5	373.8	377.7
Third	171.5	252.2	323.1	369.2	375.0
Fourth	168.0	247.0	317.8	364.6	372.0
Fifth	164.5	241.9	312.4	360.0	368.9
Tenth	147.5	217.3	286.3	337.6	352.6
Twentieth	116.9	172.6	236.1	293.1	319.1
Thirtieth	91.1	134.7	189.6	247.3	281.6

WTP for the first, second and further 1-year increases in remaining LE at ages 0, 20, 40, 60 and 80 years. The LE remaining at these ages in the baseline simulation is 78.9, 59.0, 39.5, 21.7 and 8.4 years. Values are given in US$1,000.

As above, the WTP for improvements increases with age so that the gains from slowing aging are greater for the old. According to Table 3, the value of delaying aging rises as the average age of society increases, leading to a shift in the diseases that the medical system should focus on. This is consistent with the argument of a fourth stage of Omran’s epidemiological transition (‘the age of delayed degenerative diseases’)^22,23^.

### Reversing aging (the Wolverine case)

A hypothetical alternative to the Peter Pan scenario is a reversal of aging, in which biological damage is repaired rather than slowed. For our literary reference we turn to the Marvel character Wolverine^24^ and his daughter X-23, who both possess a healing factor enabling body tissue to be regenerated. Recent advances have shown that such regeneration is possible in mice and humans^25,26^.

We capture this by assuming a one-time intervention at age 65 that rewinds an individual’s biological clock back to a specific age, *Z*. Supplementary Table 1 reports the WTP for such a reversal in which gains are once more by 1-year increases in LE; for example, in the row starting ‘First’, WTP at 0 years is US$103,500 for a first reversal in aging at age 65 that increases LE at birth from 78.9 to 79.9 years.

Reversing aging sounds more dramatic than slowing aging, but the differences in our model are subtle. This is because we assume that aging slows down over the entire adult life whereas a reversal occurs only at age 65. For this reason, the WTP in the Peter Pan case is greater than for the Wolverine case at younger ages and the WTP rises faster with age under the Wolverine case. This effect is further enhanced because reversal leads to a relative improvement of health at older ages, meaning that these years become more valuable as relatively more consumption is allocated to them.

### Targeting aging versus single diseases

The results in the Peter Pan and Wolverine cases suggest that the gains to slowing or reversing aging are substantial. This raises two further questions. How much can aging be realistically slowed? And how does the WTP for slowing aging compare to that for the reduction or eradication of specific diseases? In this section, we explore these questions with reference to metformin, a drug prescribed for type 2 diabetes that is considered to produce ‘protective effects against several age-related diseases’^27^. We do so by utilizing the results of ref. ^28^ (based on a study of 41,204 men with diabetes with an average age of 75), which provides detailed year-by-year estimates of the effect of metformin on the incidence of various age-related comorbidities.

Two features of our focus on both metformin and the results of ref. ^28^ should be emphasized. The first is that the efficacy of metformin awaits confirmation from large sample trial data such as from the Targeting Aging with Metformin (TAME) trial. Our calculated results will naturally differ if such results lead to different estimates than in ref. ^28^. The second is that the key results of this section are valid for any intervention, clinical or otherwise, that attenuates the effect of aging. For instance, education is widely seen to impact health outcomes and could be considered in exactly the same way as metformin in our simulations. The case of education shows not just the relevance of non-clinical interventions but also that interventions can occur across the life course.

For our purposes, ref. ^28^ provides estimates of a set of factors $0 \le \lambda _{a,i} \le 1$ that measure the reduction in the incidence of disease (*i*) after a year of treatment. Denoting the incidence of disease in the absence of metformin by $\pi _{a,i}$ and the same incidence when taking metformin by $\pi _{a,i}^ \ast$ the factors satisfy $\pi _{a,i}^ \ast = \lambda _{a,i}\pi _{a,i}$. If $\lambda _{a,i} = 1$ then metformin has no effect and if $\lambda _{a,i} = 0$ the disease is eradicated. In our case, the factors after 5 years of treatment are 0.52 for dementia, 0.33 for cardiovascular diseases, 0.32 for cancer, 0.29 for depression and 0.58 for frailty-related diseases. We use the Global Burden of Disease dataset^2^ to identify the number of US deaths and years lost to illness due to each of these age-related morbidities, and adjust them downwards by the $\lambda _{a,i}$ factors. The WTP for metformin consists of two components, representing gains to mortality and the health benefits arising from reduced incidence of disease.

There are two reasons to expect large gains when comparing metformin to single disease treatments. The first is the rising prevalence of age-related comorbidities, which makes targeting aging valuable as the impact will be felt across multiple diseases (see ref. ^29^). The second is synergies between diseases: reducing the incidence of any given disease has more impact on LE and health when the incidence of other diseases is also reduced (the competing risks argument^30^). In many ways, treatments that target aging are more similar to drugs that save lives at younger ages and promote longer spells of healthy life, rather than treatments aimed at extending lifespan for shorter periods of time in poor health.

We make three assumptions regarding the age at which treatment starts: 75 (the average age of participants in the study), 65 (all participants are over 65) and 50 years. As ref. ^28^ only includes men over the age of 65 with diabetes, the $\lambda _{a,i}$ factors may not accurately capture the impact of metformin on women, individuals who do not have diabetes, or those aged under 65. Metformin may also have less of an impact at higher ages^31^. The WTP calculations we present are broadly linear in the $\lambda _{a,i}$ factors so it is relatively easy to scale the gains up or down. For example, if the impact for individuals without diabetes is only 10% of that for those with diabetes, then multiplying the WTP by 0.1 gives an appropriate estimate of the gains.

Based on ref. ^28^, metformin has a sizable effect on LE. For the case in which treatment starts at age 75, LE at birth rises by 2.9 years, at 20 years rises by 3.0 years, at 40 years rises by 3.0 years, at 60 years rises by 3.3 years, and at 80 years rises by 4.3 years. The additions to remaining HLE vary from 1.7 to 2.5 years.

**Fig. 2:**
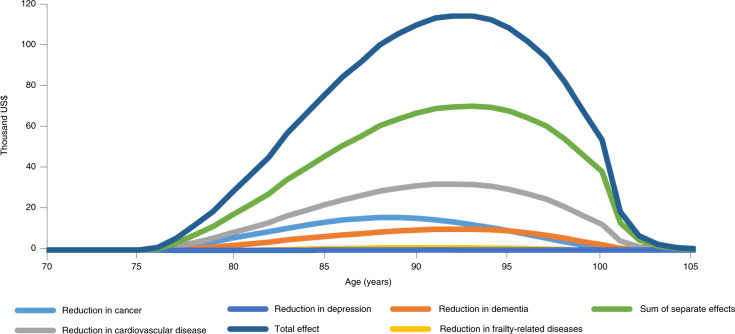
WTP by year of life for metformin treatment started at age 75. The value for each year (by age) of improvements in the incidence of various diseases under simulated impact of metformin. Sum of separate effects, the total of each individual effect; Total effect, the overall value for each year of health improvements attributed to metformin. Solid lines represent WTP for each of the five comorbidities separately. Source data

Supplementary Table 2 shows that the estimated benefits of metformin are substantial, often matching or exceeding those from the complete eradication of cancer, dementia or cardiovascular diseases. Figure 2 breaks down the WTP for metformin, starting at 75, by year of life in which the benefits occur. The total WTP for metformin significantly exceeds the sum of the separate effects due to metformin’s beneficial impact on competing risks. The magnitude of these aggregation and complementarity effects increases with the number of diseases under consideration. Although Fig. 2 focuses only on non-communicable diseases, extending the analysis to include infectious diseases such as Covid-19, whose mortality rises with age, will increase the estimates even further.

### Aggregate gains

We now shift from calculating individual gains to the total gains aggregated across all ages in society, as well as including the benefits to as yet unborn generations^8^. Focusing on the aggregate WTP reveals a powerful additional dynamic at work. Slowing down aging leads to a population that is on average older and larger (as more people live for longer), both of which increase the aggregate WTP for further improvements. This creates a virtuous circle around delaying aging; the better that society ages, the more valuable any further improvements. To calculate this aggregate WTP, we sum the age-specific individual WTPs from the Peter Pan scenarios using the latest US Census Bureau data on the population, its age structure and birth rates. For consistency, we measure improvements in terms of step increases in LE achieved by adjusting the speed of aging.

Based on Table 3 (first row) and current census data, the total WTP for a 2017 slowdown in aging leading to a 1-year increase in LE is US$37.6 trillion (US$29.7 trillion for those alive in 2017, US$7.9 trillion for those not yet born). The corresponding number for a 10-year increase in LE is US$366.8 trillion (split US$291.9 trillion, US$74.8 trillion). Based on a 2% interest rate, the value of this 10-year increase is US$7.2 trillion at an annual rate (or 33.6% of 2019 GDP). These calculations abstract from the diversity of health across the US population^32,33^, so they are likely to underestimate the aggregate gains from delaying aging.

The value of a further delay in aging in 2050 is shown in Table 4 (fourth to sixth row). These depend on the individual WTPs for a second incremental slowing of aging (for example, Table 3, second row), as well as the 2050 projected population age structure. To obtain estimates of the latter, we start from the 2017 population and forecast forward using current birth and mortality rates from 2017, adjusted for the assumed initial improvement in aging and setting net immigration to zero.

**Table 4:** Society WTP for successive slowdowns in aging

	Increase in remaining LE (years)				
	1	2	3	5	10
Society WTP for first slowdown in aging in 2017					
Living population	29.7	59.3	88.8	147.4	291.9
Unborn generations	7.9	15.6	23.3	38.5	74.8
Total	37.6	75.0	112.1	185.9	366.8
Society WTP for second slowdown in aging in 2050					
Living population	30.0	60.3	90.7	152.1	307.9
Unborn generations	6.8	13.5	20.1	32.9	62.4
Total change	36.9	73.8	110.8	185.0	370.3
Change in society WTP from 2017 to 2050					
Change in individual WTP	−0.05	−0.20	−0.50	−1.63	−8.82
Between first and second slowdown of aging					
Change in society WTP from	0.11	0.48	1.13	3.41	15.98
Increase in population due to slowdown of aging					
Change in society WTP from	0.26	0.69	1.29	2.92	8.77
Independent changes in population					
Change in society WTP of	−1.03	−2.10	−3.23	−5.62	−12.47
Unborn generations					
Total change	−0.70	−1.14	−1.31	−0.91	3.47

The aggregate WTP for the 2017 and 2050 delays in aging are of similar magnitude. For smaller improvements in LE, the WTPs for the second wave are worth slightly less (approximately 1–2%) but for larger improvements slightly more (approximately 1%). Table 4 provides a breakdown of the factors driving the change in the aggregate WTP. One reason that the aggregate WTP changes between the two rounds is due to changes in individual age-specific WTPs. As shown in Table 3, the WTP for further delays in aging declines at each age and this lowers the 2050 aggregate WTP. This effect is shown in the first line of the breakdown (Table 4), in which a 1-year increase in LE decreases the aggregate WTP by US$45.8 billion. The aggregate WTP also changes between the two waves of aging improvements because of changes in population. Independent of the delays in aging that we model, there is expected to be an increase in the average age of the US population of 4.0 years between 2017 and 2050 and (assuming zero net immigration) a decrease in population size by 1.6 million people. The increase in average age boosts the aggregate WTP (delaying aging is more valuable for the old) while a shrinking population lowers it (aggregation occurs over fewer people). The second row of the deconstruction shows that the aging effect dominates, so the combined impact is positive and raises the aggregate WTP by US$113.7 billion in the case of a 1-year increase in LE.

Additional changes in the age structure are induced by the assumed delay in aging. This leads to more people alive at older ages and in better health in 2050, raising the aggregate WTP. Table 4, third row, shows that this is worth US$256.7 billion for the case of a 1-year in crease in LE and US$8.8 trillion for a 10-year increase in LE. Importantly, the size of this channel increases faster than the gains to LE. Improvements in LE have a disproportionate impact on the size and age of the older population, so this induced population change increases rapidly in response to improvements in aging. It is this that produces the virtuous circle through aggregation.

For small improvements in LE, the negative effects of declining individual WTP and fewer births are greater than the positive effects from changes in population structure. As a consequence, the aggregate value of gains to aging declines. Closer examination suggests that this virtuous circle remains true even for the case of small gains in aging. Focusing on the two factors that are endogenous to our model (changes in the individual WTP and induced population changes) reveals their sum to be always positive, reflecting an aggregate virtuous circle.

The second reason that the virtuous circle exists for even small delays in aging is connected to whether the WTP for gains to LE are really declining at the individual level. Throughout, we have focused on measuring improvements in health, longevity and aging by focusing on 1-year step increases in LE. However, one reason that the individual WTPs for Peter Pan decline in response to further delays in aging is that each 1-year increase in LE represents a smaller percentage increase in LE and larger proportional changes in mortality rates. If instead we focus on percentage improvements in aging (for example, a 1% slowdown in biological aging rather than a slowdown generating a 1-year increase in LE) then we have increasing WTP at an individual level. In other words, measurement of aging biologically rather than chronologically leads to increasing returns for aging at the individual level, feeding into even greater increasing returns at the aggregate level.

## Conclusion

The economic value of gains from targeting aging are large because delaying aging produces complementarities between health and longevity, affect a large number of diseases due to the rising prevalence of age-related comorbidities, and create synergies arising from competing risks. Crucially, delaying aging leads to a virtuous circle in which slowing aging begets demand for further slowing in aging. This virtuous circle arises because society’s gains from delaying aging rise with the average age of society, increase with the quality of life in old age, and depend on the number of older people. This provides a distinctive dynamic to targeting aging compared to treatments aimed at specific diseases, in which gains diminish once successful treatments are discovered.

Our estimates are larger than those in ref. ^29^, which calculates a slowdown in aging producing a 2.2-year increase in LE as worth US$7.1 trillion to those aged over 51. This case is closest to our 2-year increase in Table 4. Adjusting for differences in chosen discount rates and VSLs and restricting our gains to the over 50 s only leads to an estimate of the aggregate gains as worth US$21 trillion. The remaining differences are attributable to ref. ^29^ assuming a phased rather than immediate improvement in aging. Although differences remain, the most important insight is that their different approach (using an empirical microsimulation model based on US individual data) arrives at similar very large estimates for the value of delaying aging.

Our estimates abstract from both inequalities in health and income. Allowing for health inequalities is likely to increase the value of the aggregate gains, but introducing income inequality raises important distributional issues. Our estimates suggest that treatments that target aging are extremely valuable. If the cost of such treatments is low then access to them will be widespread. If, however, the costs are high then issues of access and redistribution will become important. What is clear from the magnitude of the potential values outlined in our simulations is the need to ensure widespread access if the full value of these social gains is to be realized.

## Methods

### Economic model

At the heart of our model is lifetime expected utility from the perspective of age *a*, given by

$$\mathop {\int }\nolimits_a^\infty H(t)u(c(t),l(t))S^ \ast (t,a)e^{ - \rho (t - a)}dt$$

where *H*(*t*) denotes health at age *t*, $u(c(t),l(t))$ the utility function (which depends on consumption *c*(*t*) and leisure *l*(*t*)), $S^ \ast \left( {t,a} \right)$ is the survival rate from age *a* to *t*, and *ρ* is the subjective discount rate determining the weight individuals give to the future. As shown previously^8^, assuming an optimizing agent gives the value of a life year at age *t* as $v\left( t \right) = w\left( t \right)(T - l\left( t \right)) - c\left( t \right) + u^\prime (c\left( t \right),l\left( t \right))/u_c^\prime$, where *w*(*t*) is the wage rate, *T* – *l*(*t*) is working hours and $u_c^\prime$ is the marginal utility of consumption, $\partial u(.)/\partial c$.

The value of a life year therefore depends on two items: a term reflecting the value of utility gained that period from consumption and leisure, $u^\prime (c\left( t \right),l\left( t \right))/u_c^\prime$; and a term reflecting savings. Years in which savings are positive are given a higher value as they provide financing for consumption at other points in life. An important feature of this model is that the value of life is substantially higher than the value of income earned over a life. That is because leisure itself has a value and the wage at any age provides a way to value this, even if an individual is not working.

Using this approach, the value of life at age *a* is $V\left( a \right) = \mathop {\int }\nolimits_a^\infty v\left( t \right)e^{ - r\left( {t - a} \right)}S^ \ast \left( {t,a} \right)dt$, where *r* is the real return the individual earns on their assets. Based on this formula^8^, we show that WTP at age *a* for improvements in longevity in response to changes in medical knowledge $(\zeta )$ is

$$\mathop {\int }\nolimits_a^\infty v\left( t \right)S(t,a)\frac{{\partial \log S(t,a)}}{{\partial \zeta }},$$

while the WTP for improvements in health is

$$\mathop {\int }\nolimits_a^\infty \frac{{H_\varsigma ^\prime (t)}}{{H(t)}}\frac{{u(c(t),l(t))}}{{u_c^\prime }}S(t,a)dt,$$

where $S(t,a) = S^ \ast (t,a)e^{r(a - t)}$, the discounted survival function.

Following ref. ^8^, we assume that utility depends on a composite *z* of consumption and leisure such that $z = \left[ {\phi c^{1 - \frac{1}{\eta }} + \left( {1 - \phi } \right)l^{1 - \frac{1}{\eta }}} \right]^{\frac{\eta }{{\eta - 1}}}$, where *η* denotes the elasticity of substitution between consumption and leisure, the willingness of the individual to trade off consumption against leisure^34^. The utility function is

$$u\left( z \right) = \frac{{z^{1 - 1/\sigma } - z_0^{1 - 1/\sigma }}}{{1 - 1/\sigma }},$$

where *z*_0_ (as in ref. ^35^) is a normalization capturing an individual’s attitude towards life versus non-existence. The parameter *σ* is the intertemporal elasticity of substitution (IES) that plays a key role in the model as it captures the willingness of the individual to reallocate consumption across time periods. The higher the IES the more an individual is concerned about total life consumption, and the lower the IES the more they are concerned about per-period consumption.

Our model follows a three-stage life, of childhood and education, work and then retirement. We assume that adulthood begins at age 20 and that consumption during childhood is financed by parents. We assume an initial wage that is constant between 20 and 25 years and then starts to rise with age such that $w(a)/w\left( {20} \right) = \gamma \log a$ until retirement at *a* = *R*, with *γ* reflecting the degree to which wages rise with experience. For *a* > *R* we set wages equal to $w\left( a \right) = {\mathrm{{\Psi}}}\left( a \right)w(R)$. In the case in which ${\mathrm{{\Psi}}}\left( a \right) = 1$, the wage post retirement is equal to the retirement value and does not decline (consistent with a previous model^36^). The case of ${\mathrm{{\Psi}}}\left( a \right) < 1,\,a > R$ is consistent with previous studies^37,38^ and we allow for this with the interpretation offered by ref. ^36^ that the discount reflects a shift to part time work paying a lower salary. The post-retirement wage falls in line with health with elasticity *ξ*.

### Health and mortality

We use a Gompertz equation for mortality, in which imposing a compensating effect of mortality gives the restricted expression $\mu \left( a \right) = Me^{\beta (a - T)}$. We set *T* = 97.6 and *M* = 0.3319 based on cross-country evidence^39^, and then calibrate *β* = 0.0966 so that LE at birth matches that in the US for 2018 (78.9 years).

For health we follow refs. ^13,40^ and assume at age *a* that an individual has disabilities given by $D\left( a \right) = E + B^{ - \mu a}$ and health at age *a* is $H\left( a \right) = [D(0)/D(a)]^\alpha$. We impose a compensating effect of morbidity using the restriction $B = D^ \ast e^{ - \mu T}$ so that $D\left( a \right) = E + D^ \ast e^{\mu (a - T)}$. For calibration purposes we use the results of previous studies^20,41^: *E* = 0.0821, *B* = exp(−0.504), *α* = 0.34. We choose *µ* to match US HLE in 2018 of 68.5 years (World Bank data), where HLE is defined by $\mathop {\smallint }\limits_0^\infty H\left( t \right)S^ \ast \left( {t,a} \right)dt$.

For Peter Pan we assume that aging is captured by a frailty index $F\left( a \right) = \theta e^{\delta a}$ and impose a compensating effect such that $\theta = F^ \ast e^{\delta T}$ so that $F\left( a \right) = F^ \ast e^{\delta (a - T)}$. We assume that the disability index is given by $D\left( a \right) = E + BF(a)^\psi$ and mortality by $\mu \left( a \right) = M^ \ast F(a)^\lambda$, pinning down a relationship between our earlier parameterization and this common factor. For our Peter Pan simulations we vary *δ*, *µ* and *T* in order to simultaneously elongate both the health and survival functions.

For our Wolverine simulations we introduce a repair function $R\left( x \right) = I(a)e^{ - \delta Z}$ such that $I\left( a \right) = 1$ for *a* ≥ *x*, and 0 otherwise. Multiplying our frailty index *F*(*a*) by *R*(*x*) gives the function $\theta e^{\delta (a - Z)}$ for *a* ≥ *x*, and $e^{\delta a}$ otherwise. Therefore, the effect of the repair is to reset a person’s biological clock by *Z* years. Modeling the Wolverine scenario requires more auxiliary assumptions than our other simulations, including at what age a reset is made, how many times it can be applied, and whether it suffers from diminishing effects. We focus on a one-time application to show theoretical differences with Peter Pan (if continually applied then Wolverine approaches Peter Pan). The earlier the age at which the reset is applied the smaller the gains achieved, so we focus on a reset at 65 years. Results with resets at different ages lead to different economic values but do not change the qualitative nature of our results.

Details of our model calibration are shown in Supplementary Table 3.

### Aggregation

The aggregate WTP based on the age distribution of the population in 2017 is

$$\mathop {\int }\nolimits_0^\infty WTP\left( a \right)_{2017}N\left( {a,2017} \right)da + WTP\left( 0 \right)_{2017}\mathop {\int }\nolimits_0^\infty B\left( {2017 + t} \right)e^{ - rt}dt,$$

where $WTP\left( a \right)_{2017}$ is the WTP at age *a* for the initial improvement in aging, $N\left( {a,2017} \right)$ is the number of people of age *a* in 2017 and $B\left( {2017 + t} \right)$ is the number of births in the year 2017 + *t*. A similar expression is used to calculate the aggregate WTP in year 2050 for a second improvement in aging. The difference between the two aggregate WTPs is given by the following:

$$\mathop {\int }\nolimits_0^\infty WTP\left( a \right)_{2050}N^ \ast \left( {a,2050} \right)da + WTP\left( 0 \right)_{2050}\mathop {\int }\nolimits_0^\infty B\left( {2050 + t} \right)e^{ - rt}dt$$

$$- \mathop {\int }\nolimits_0^\infty WTP\left( a \right)_{2017}N\left( {a,2017} \right)da + WTP\left( 0 \right)_{2017}\mathop {\int }\nolimits_0^\infty B\left( {2017 + t} \right)e^{ - rt}dt$$

$$= \mathop {\int }\nolimits_0^\infty (WTP\left( a \right)_{2050} - WTP\left( a \right)_{2017})N\left( {a,2017} \right)da$$

$$+ \mathop {\int }\nolimits_0^\infty WTP\left( a \right)_{2050}(N^ \ast \left( {a,2050} \right) - N\left( {a,2050} \right))da$$

$$+ \mathop {\int }\nolimits_0^\infty WTP\left( a \right)_{2050}(N\left( {a,2050} \right) - N\left( {a,2017} \right))da$$

$$\begin{array}{l}+ WTP\left( 0 \right)_{2050}\mathop {\int }\nolimits_0^\infty B\left( {2050 + t} \right)e^{ - rt}dt \\- WTP\left( 0 \right)_{2017}\mathop {\int }\nolimits_0^\infty B\left( {2017 + t} \right)e^{ - rt}dt\end{array}$$

where $N^ \ast \left( {a,2050} \right)$ is the number of people of age *a* in 2050 allowing for the impact of the initial improvement in aging and $N\left( {a,2050} \right)$ is the number of people of age *a* in 2050 in the baseline projection without the improvement in aging.

### Reporting Summary

Further information on research design is available in the Nature Research Reporting Summary linked to this article.

## Supplementary information

Supplementary Information Supplementary Tables 1–3.

Reporting Summary

## Data Availability

The data for the incidence of disease were taken from the Global Burden of Disease dataset (http://ghdx.healthdata.org/gbd-2019). The US population Census data were taken from https://www.census.gov/programs-surveys/popproj/data/datasets.html.

## Source data

Source Data Fig. 1 Underlying data for chart.
Source Data Fig. 2 Underlying data for chart.

## Code availability

All simulations were performed using MATLAB version 9.6.0.1072779 (R2019a). All codes are available upon request.
