# Supplementary material for: The economic value of targeting aging
Source: Nat Aging. 2021 Jul 5;1(7):616–23. doi: 10.1038/s43587-021-00080-0 (PMC10154220; doi:10.1038/s43587-021-00080-0)
Supplement: Supplementary file 1 — Supplementary Tables 1–3. [file 43587_2021_80_MOESM1_ESM.pdf]

nature portfolio

https://doi.org/10.1038/s43587-021-00080-0

Supplementary information

The economic value of targeting aging

In the format provided by the authors and unedited

Supplementary Table 1: Wolverine: Willingness to Pay (WTP) for reversing aging at age 65 ($ thousand)

	Age at which WTP calculated				
	0	20	40	60	80
1st +1 year	103.5	153.8	228.6	339.6	372.9
2nd +1 year	103.0	153.1	227.5	338.2	373.4
3rd +1 year	102.5	152.3	226.4	336.7	373.4
4th +1 year	102.0	151.5	225.2	335.1	373.0
5th +1 year	101.4	150.7	224.0	333.4	372.4
10th +1 year	98.2	145.9	216.9	323.9	366.0
20th +1 year	90.1	133.9	199.4	299.9	343.1
30th +1 year	80.7	120.0	179.0	272.3	313.6

Notes: The rows show the Willingness to Pay (WTP) for a reversal of aging at 65 that leads to the 1st, 2nd, 3rd ... 30th one-year increase in remaining life expectancy (LE) at ages 0, 20, 40, 60, 80. The LE remaining at these ages in the baseline simulation is 78.9, 59.0, 39.5, 21.7 and 8.4 years.

Supplementary Table 2: Willingness to pay (WTP) for metformin treatments and disease eradications ($ thousand)

	Age at which WTP calculated				
	0	20	40	60	80
Metformin started at age 75	188.4	281.4	429.2	697.7	1365.7
Metformin started at age 65	365.9	546.3	833.2	1354.6	1835.0
Metformin started at age 50	712.5	1064.0	1622.8	2204.1	2336.7
Eradication of cancer	361.8	528.5	749.4	840.6	436.7
Eradication of dementia	68.4	102.0	155.6	247.2	397.1
Eradication of cardiovascular disease	439.7	651.9	928.7	1139.3	1123.9

Notes: The life expectancy remaining at ages 0, 20, 40, 60, 80 when not taking metformin is 78.9, 59.0, 39.5, 21.7 and 8.4 years. At these ages when taking metformin, it is 82.7, 62.9, 43.3, 25.2 and 3.0 years.

Supplementary Table 3: Calibration

Parameter	Value	Source
$1/\sigma$	1.5	[34] Table 3
η	1.509	[34] Table 4
ϕ	0.224	[34] Table 4
$w(20)$	6.98	VSL at birth $11.5million [14]
R	65	Retirement Age
γ	1.35	[36] Figure 1
$\Psi(R)$	0.68	[36] Table 1
ξ	1.75	[36] Figure 1
$r = \rho$	0.02	[8]
M	0.3319	[39] Table 2 average across male and female
T	97.6	[39] Table 2 average across male and female
β	0.0966	Life Expectancy (LE) at birth 78.9 years
E	0.0821	[20] Table 3 average across male and female
B	exp(-0.504)	[20] Table 5
α	0.34	[40],[41]
μ	0.0516	Healthy Life Expectancy (HLE) at birth is 68.5 years
z_0/z	0.1	[8]
