# Supplementary material for: The economic value of targeting aging
Source: Nat Aging. 2021 Jul 5;1(7):616–23. doi: 10.1038/s43587-021-00080-0 (PMC10154220; doi:10.1038/s43587-021-00080-0)
Supplement: Supplementary file 2 — Reporting Summary [file 43587_2021_80_MOESM2_ESM.pdf]

nature research

Corresponding author(s): Andrew J Scott

Last updated by author(s): Apr 12, 2021

Reporting Summary

Nature Research wishes to improve the reproducibility of the work that we publish. This form provides structure for consistency and transparency in reporting. For further information on Nature Research policies, see our Editorial Policies and the Editorial Policy Checklist.

Statistics

For all statistical analyses, confirm that the following items are present in the figure legend, table legend, main text, or Methods section.

n/a	Confirmed	
☒	☐	The exact sample size (*n*) for each experimental group/condition, given as a discrete number and unit of measurement
☒	☐	A statement on whether measurements were taken from distinct samples or whether the same sample was measured repeatedly
☒	☐	The statistical test(s) used AND whether they are one- or two-sided<br>*Only common tests should be described solely by name; describe more complex techniques in the Methods section.*
☒	☐	A description of all covariates tested
☒	☐	A description of any assumptions or corrections, such as tests of normality and adjustment for multiple comparisons
☒	☐	A full description of the statistical parameters including central tendency (e.g. means) or other basic estimates (e.g. regression coefficient) AND variation (e.g. standard deviation) or associated estimates of uncertainty (e.g. confidence intervals)
☒	☐	For null hypothesis testing, the test statistic (e.g. F, t, r) with confidence intervals, effect sizes, degrees of freedom and P value noted<br>*Give P values as exact values whenever suitable.*
☒	☐	For Bayesian analysis, information on the choice of priors and Markov chain Monte Carlo settings
☒	☐	For hierarchical and complex designs, identification of the appropriate level for tests and full reporting of outcomes
☒	☐	Estimates of effect sizes (e.g. Cohen's *d*, Pearson's *r*), indicating how they were calculated

Our web collection on statistics for biologists contains articles on many of the points above.

Software and code

Policy information about availability of computer code

Data collection	No software was used.
Data analysis	MATLAB Version: 9.6.0.10 7 2779 (R2019a)

For manuscripts utilizing custom algorithms or software that are central to the research but not yet described in published literature, software must be made available to editors and reviewers. We strongly encourage code deposition in a community repository (e.g. GitHub). See the Nature Research guidelines for submitting code & software for further information.

Data

Policy information about availability of data

All manuscripts must include a data availability statement. This statement should provide the following information, where applicable:

- Accession codes, unique identifiers, or web links for publicly available datasets
- A list of figures that have associated raw data
- A description of any restrictions on data availability

We provide a full data availability statement in the manuscript.

Field-specific reporting

Please select the one below that is the best fit for your research. If you are not sure, read the appropriate sections before making your selection.

☐ Life sciences ☒ Behavioural & social sciences ☐ Ecological, evolutionary & environmental sciences

For a reference copy of the document with all sections, see nature.com/documents/nr-reporting-summary-flat.pdf

Behavioural & social sciences study design

All studies must disclose on these points even when the disclosure is negative.

Study description	Quantitative numerical simulation of theoretical model. Our model is based around a series of mathematical equations described in the Methods section. This model is calibrated as shown in Supplementary Table 3. With this calibration we can then use Matlab to solve for consumption and leisure and other key variables and create our simulated data. We can then use this simulated data to estimate the Willingness to Pay (WTP) for various changes in health and life expectancy. The WTP formulas are listed in the Methods section.
Research sample	The majority of the data is simulated data (see above). The exceptions are the data for:<br>- The effect of metformin interventions. As listed in the paper these are from C.-P. Wang, C. Lorenzo, S. L. Habib, B. Jo, and S. E. Espinoza. Differential effects of metformin on age related comorbidities in older men with type 2 diabetes. Journal of Diabetes and its Complications, 31(4):679–686, Apr. 2017 and the data was provided to us by Prof.Wang<br>- The data for the incidence of disease taken from Global Burden of Disease Dataset http://ghdx.healthdata.org/gbd-2019<br>- And the US population data taken from https://www.census.gov/programs-surveys/popproj/data/datasets.html
Sampling strategy	There is no statistical inference in our study so no need to sample. We select the US as an example country and calibrate data around averages.
Data collection	As described above the only primary data in our study was created using our own simulations using code so is not collected in a traditional sense. Other data is secondary taken from the US census online dataset or the GBD online dataset. So collection involves merely downloading in terms of our contribution but the Census and GBD obviously provide extensive guidance as to their own data collection. The only other data was that provided by Prof Wang which is not data per se but the results of his analysis of data e.g. how metformin impacts disease incidence. So no individual data was collected and no primary real world data constructed.
Timing	As described above this question is not pertinent. We are not collecting or creating primary datasets. We use the latest version of the census data and the GBD but this is the version that is published and referred to (2017 Census, 2019 GBD).
Data exclusions	Again, because no primary data set was created this question is not relevant. Data use is explained in the paper and all relevant data was used.
Non-participation	There were no participants and so none dropped out or declined.
Randomization	If randomization refers to RCT then it isn't relevant here. As explained above we simulate our data so there is no sampling. Further because the model is simulated there are no physical interventions requiring RCT. Given our model we can calculate precisely the numerical answers.

Reporting for specific materials, systems and methods

We require information from authors about some types of materials, experimental systems and methods used in many studies. Here, indicate whether each material, system or method listed is relevant to your study. If you are not sure if a list item applies to your research, read the appropriate section before selecting a response.

Materials & experimental systems

n/a	Involved in the study
☒	☐ Antibodies
☒	☐ Eukaryotic cell lines
☒	☐ Palaeontology and archaeology
☒	☐ Animals and other organisms
☒	☐ Human research participants
☒	☐ Clinical data
☒	☐ Dual use research of concern

Methods

n/a	Involved in the study
☒	☐ ChIP-seq
☒	☐ Flow cytometry
☒	☐ MRI-based neuroimaging
